# Treatments’ Outcomes of Patients Suffered from Trigeminal Neuralgia in Kerman, Iran

**Published:** 2014-09

**Authors:** Javad Faryabi, Maryam Joolhar

**Affiliations:** a Dept. of Oral & Maxillofacial Surgery, School of Dentistry, Member of Oral and Dental Diseases Research Center, Kerman University of Medical Sciences, Kerman, Iran.; b General Dentist, Private Practice, Tehran, Iran.

**Keywords:** Trigeminal neuralgia, Drug therapy, Microvascular decompression

## Abstract

**Statement of the Problem:** Trigeminal neuralgia (TN) presents with a shooting pain in maxillofacial region which compels the involved patients to visit many medical and dental physicians to relieve the pain. Hence, assessing the outcomes of different treatment modalities may help the patients and their clinicians choose a suitable practical method of treatment.

**Purpose:** The study was aimed to evaluate the outcomes of different treatments of TN and to determine which surgical or non-surgical treatment is better for controlling the pain.

**Materials and Method:** This study included 42 patients with trigeminal neuralgia. A questionnaire was completed for each patient in order to evaluate the pain control status of patients’ treatment with non-surgical (pharmaceutical) and surgical modalities. The questionnaire consisted of questions on an datasheet , concerning the duration of involvement with the condition, type and dose of the medication(s) used, the surgical technique administrated, patient satisfaction with the treatment modality and the intensity and frequency of the pain if present. The purpose of the study and the necessary information regarding the questions of the data sheet was given in detail to the patients for a careful completion of the questionnaires. Data was analyzed by adopting t-test using SPSS software.

**Results:** The results showed that the patients treated with pharmaceutical modalities had relatively lower improvement rate than those treated with surgery combined with medication (*p*< 0.035). Carbamazepine was the most consumed medication for pain control and the microvascular decompression was the most performed surgical method in patients.

**Conclusion:** Although medication therapy alone was less effective than surgery combined with medication, both treatment modalities were significantly effective in controlling the pain of patients.

## Introduction

Trigeminal neuralgia (TN) is a known medical condition and its published reports date back to the first century. The condition is manifested as a shooting pain in the oral and maxillofacial region [1-2]. The sudden shock-like pain starts by mild stimuli such as tooth brushing, shaving, or washing of the face [3-4]. Usually, the right side of the face is affected twice that of the left side [5]. Patients may visit several physicians due to the excruciating pain that they experience and usually dental practitioners are the first to consult after burden and the onset of recurrent pain episodes [6]. However, with a detrimental effect of this condition on the patients’ quality of life, they refer to several physicians since a complete recovery from the condition may not usually happen [2]. On the other side, dealing with many unsatisfied and suffering patients, this condition is considered as a serious clinical challenge for their clinicians [7].

Drug therapy, with Carbamazepine as the first choice, is usually considered the first line of non-surgical approach for the treatment trigeminal neuralgia [2]. However, other anticonvulsive drugs such as Phenytoin, Lamotrigine, Oxcarbamazepine, Topiramate, and Baclofen are also used to prevent acute attacks of the condition [8-10]. Since numerous patients do not respond effectively to drug therapy [8] and these medications have adverse effects when used in long term (such as drowsiness, nausea, confusion, ataxia or lack of balance, and rarely irreversible aplasic anemia) [11], various surgical techniques have been proposed for this burden. The surgery methods include microvascular decompression (MVD) of trigeminal ganglion [12], gamma knife radio-surgery [13], surgery of the trigeminal ganglion without decompression [14], and peripheral injection of alcohol [15]. Moreover, patients may benefit from homeopathy [8] and particular dental treatment modalities. The dental treatment can be a periapical and bone surgery such as curettage of alveolar bone cavities in these patients namely, Neuralgia-Inducing Cavitational Osteonecrosis (NICO) [13, 16]. In some instances, patients had extraction of some of their teeth (even all teeth) to alleviate the TN pain, though without any success [3]. While some surgical treatments have been unable to relieve pain, this study has been conducted to evaluate the efficacy of medical treatment versus surgical modalities for relieving the pain in patients suffering from TN.

## Materials and Method

42 patients with trigeminal neuralgia, who had referred to major treatment centers including private offices, state specialized treatment centers and the maxillofacial surgeons’ offices in Kerman, were recruited in this study. The demographic information of the participants was collected and then the patients were invited to the Department of Oral and Maxillofacial Surgery of the Faculty of Dentistry, Kerman University of Medical Sciences, for examination, interview and completion of a questionnaire. If a valid medical record was available, it was employed for tracking the course of the disease and for further evaluation. To be included in the study, the course of the disease should have been passed for 4 weeks (at least) from commencement of the condition, diagnosis and establishment of treatment [10].

The participants were excluded from the study if:

1. There were any doubts about the definitive diagnosis of TN, including patients with pain resulting from temporomandibular disorders (TMD) and burning mouth syndrome (BMS)   
2. If the pain had not been alleviated by the treatments rendered, based on the criteria presented by Sweet:
  - Sudden pain
  - Pain elicited by mild palpation of the face (pain at the target point)
  - Pain in the trigeminal nerve dermatome
  - Unilateral pain
  - Normal facial sensation during clinical examination [6]

In order to assess the severity of pain after treatment, visual analog scale (VAS) test was enrolled and the patients were asked to mark the level of their perceived pain on a 100 mm, non -hatched VSA scale with one end marked as “no pain” and at the other end as “worst pain imaginable” [10, 17].

The pain severity was determined by the number of attacks (frequency of attack) as Mild (0-5 times a day), Moderate (6-10 times a day), Severe (11-15 times a day), and Very Severe (more than 15 times a day) [8].

The data collected by the questionnaire consisted of patients’ age, duration of involvement with TN, the first treatment received, supplementary treatment received (if any), and severity of the present pain (mild, moderate, severe, very severe).

To verify these data, an attempt was made to access patient medical files (if available) in order to record the therapeutic course. When the type of the supplementary (particularly surgical treatment) received by the patient was unidentified, the patient was excluded from the study. All the patients participating in the study were informed about the nature of the study in details and then deliberately signed informed written consent forms.

## Results

Data were initially described by using frequency tables, graphs and central statistical parameters, including median, mean, and standard deviation.

**Type of pain**

In the present study, the most frequent type of pain was shooting pain with a frequency of 42.8%. Other types of pain are presented in Table 1.

**Table 1:** Frequency distribution of the types of pain in the patients of this study

** Type of the pain**	**Frequency**	**Frequency percentage**
Shooting	18	42.8
Electric shock	11	26.1
Burning	8	19.1
Throbbing	2	4.8
Disseminated	1	2.4
Itching	1	2.4
Dull	1	2.4
Total	42	100.0

**Initiation of the first episode of pain**

Pain had initiated suddenly and spontaneously in 22 patients (32.4%) and after tooth extraction in 8 patients (19%). Other instances included initiation of pain after eating, palpation of the area, cold water, emotional stress, toothache, and trauma which are summarized in Table 2.

**Table 2:** Frequency distribution for the instances the first episode of pain initiated

**The causes of initiation of pain**	**Frequency**	**Frequency percentage**
Sudden pain	22	52.4
After tooth extraction	8	19.0
By eating	3	7.1
By palpation	3	7.1
By cold water	2	4.8
By emotional stress	2	4.8
After toothache	1	2.4
After trauma	1	2.4
Total	42	100.0

**Duration of involvement**

Duration of involvement of 28 patients (66.7%) was less than 10 years and in 14 patients (33.3%) was more than 10 years. Other occasions are summarized in Table 3.

**Table 3:** Frequency distribution of the duration of involvement with trigeminal neuralgia in the patients in this study

**Duration of affliction (years)**	**Frequency**	**Frequency percentage**
1 month-1 year	8	19.0
1-5	13	31.0
5-10	7	16.7
10-15	8	19.0
15-20	3	7.1
20-25	2	4.8
25-30	1	2.4
Total	42	100.0

**The affected side**

The right side of the face was more involved than the left side in the present study. The right and left sides were affected in 24 (57.1%) and 17 (40.5%) patients, respectively. Bilateral involvement was identified in only one patient (2.4%).

**Use of Medication**

**Carbamazepine**

40 patients (95.2%) in the present study had taken Carbamazepine, with the routine daily dose of 200-1200 mg. The most prevalent dose was 400 mg/day in 9 patients (21.4%), followed by 600 and 800 mg/day in 8 patients (19%) each. Seven patients (16.7%) took 1200 mg/day and in 3 patients (7.1%) the dose was higher than 1200 mg/day. 

**Phenytoin**

5 patients (11.9%) had taken Phenytoin to control pain with doses of 300 mg/day in 4 patients and 200 mg/day in 1 patient (2.4%). 

**Baclofen**

8 patients (19%) had taken Baclofen; with 3 patients (7.1%) taking the highest does of 10 mg/day. The daily doses of 20 and 30 mg had been taken by 2 patients each (4.8%) and only one patient (2.4%) had taken a daily dose of 75 mg.

**Gabapentin**

Gabapentin had been taken by 4 patients (9.5%) at different doses.

**Amitriptyline**

Only 1 patient (2.4%) had taken Amitriptyline at a dose of 25 mg/day.

**Lamotrigine**

No patient had taken Lamotrigine.

**Duration of the medication therapy**

32 patients had taken medications for 1 month to 5 years (76.1%) and 7 patients (16.6%) for 5-10 years. Only 3 patients (7.1%) had taken medications for 15-20 years. 

**Surgical technique for the treatment of neuralgia**

Of a total of 42 patients, 16 patients had undergone surgery; 14 patients (87.5%) had undergone MVD, 1 patient (6.25%) had undergone cryosurgery and 1 patient (6.25%) had received Botox injection. 

**Satisfaction of patients with the treatment of neuralgia**

From the total of 42 patients, 29 patients (69%) were completely satisfied with treatments ( medication therapy, surgery-medication therapy) and ; 8 and 10 patients were fully satisfied with medication therapy and surgery, respectively. 11 patients (26.1%) were fairly satisfied; 6 patients (14.3%) were dissatisfied and 7 patients (16.66%) were very dissatisfied .Totally, 13 persons (30.95%) were dissatisfied. 

**Severity of the pain**

The pain severity, measured on VAS, was 90-100 in 11 patients (26.1%); 10-20 in 3 patients (7.1%), and 60-70 in 3 other patients (7.1%). Other pain severities were less prevalent (Figure 1 and Table 4).

A total of 13 patients (30.95%) had low pain severity and subsequently 6 (14.28%), 3 (7.14%), and 2 (4.8%) patients exhibited very severe, severe, and mild pain severity respectively.

Comparing medication therapy alone (carbamazepine) and the surgery combined with medication therapy, MVD with statistically significant differences (*p*< 0.035) (∂ =5%) revealed that fewer patients had recovered from TN by using medication therapy alone.

**Figure:**
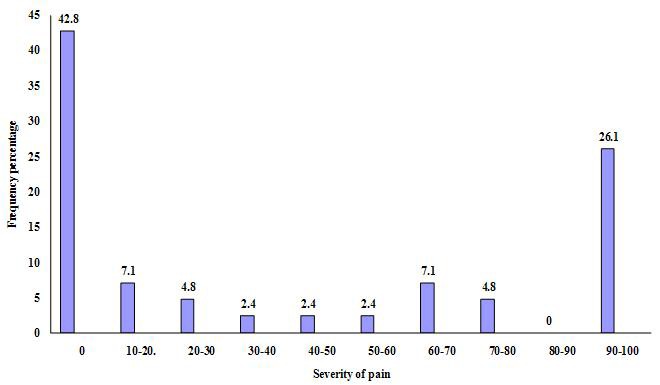
Frequency of pain severity in patients with TN after treatment

**Table 4:** Frequency distribution of pain severity in patients with NT after surgery

**** **Severity of pain**	**Surgery**				**Total**
	**Drug therapy**	**MVD**	**Cryosurgery**	**Botox**	
0	8	9	0	1	18
10-20	1	1	1	0	3
20-30	1	1	0	0	2
30-40	1	0	0	0	1
40-50	1	0	0	0	1
50-60	1	0	0	0	1
60-70	2	1	0	0	3
70-80	1	1	0	0	2
90-100	10	1	0	0	11
Total	26	14	1	1	42

## Discussion

In the present study, 18 patients (42.8%) had completely recovered from TN with the treatments they had received and 26.1% had achieved relative pain relief, consistent with the results of a study published by Scrivani et al. [6] who concluded that there were limited therapeutic choices available in most chronic neurologic pain disorders and limited response were observed with the employed treatments. Nevertheless, TN was the only disorder in which the majority of patients responded well to the drug therapy and surgical procedures and they completely relieved from pain attacks for several months or years [6].

In the present study, medication therapy resulted in pain relief in 57.67% of patients with complete pain relief in 30.75% and partial pain relief in 26.92%. Moreover, 57.14% of patients experienced pain recovery by using carbamazepine; with complete pain relief in half of these patients (28.57%) and partial pain relief in the remaining half. These findings are in consistent with the results of some previously published studies [6, 18-19]. In a study by Taylor et al. half of the patients with TN achieved recovery by drug therapy, almost 50% of the patients did not initially respond to drug therapy or had developed drug resistance and finally entered the surgical phase [18]. In a study enrolled by Scrivani et al. results showed that 50% of patients experienced proper pain relief with the use of anticonvulsive agents [6]. In a survey published by Campbell et al., it was found that Carbamazepine had a better and more appropriate effect compared to placebo in the treatment of TN and resulted in almost 58% pain relief during the first course of drug use [19].

On the other hand, the results of the present study are not in line with those of yielded by the study of Jun Sato et al. concerning the effect of medication therapy on the pain relief. In their study, 50 patients with TN were evaluated and their findings showed that Carbamazepine was effective for the treatment of TN and resulted in pain relief in 45 patients (90%). Moreover, Rockliff et al. studied the efficacy of anticonvulsive agents, especially Carbamazepine, and reported that the efficacy of the medications taken by their studied patients was 60-80% [20]. The differences in the results of these studies might probably be due to the improper management and control of anticonvulsive agents, especially Carbamazepine in their study.

Of all the patients treated with MVD surgical procedure in the present study, 85.5% achieved complete or partial recovery which is consistent with the results of previous studies. In a published study conducted by Olson et al., 93% of patients achieved complete recovery and 4% achieved partial recovery after MVD surgical procedure [12]. Scrivani et al. reported that surgical techniques were significantly effective and well tolerated by studied patients [6].

In a research by Neto et al., smaller size of the foramen rotundum and foramen ovale on the right side were reported as the etiologic factors for TN. They reported a higher incidence of the condition on the right side. Their results showed that 83% of patients experienced recovery with the use of MVD surgical technique and the rest (17%) did not achieve pain relief and recovery with the surgical technique. They concluded it was attributed to other etiologic factors rather than pressure on the nerves and inappropriate treatment [5].

In a study enrolled by Reuelta-Gutierrez et al., presence of blood vessels such as superior cerebellar artery and its pressure on the trigeminal nerve was not detected in 3.1-17% of patients and standard MVD surgical technique was introduced as a safe and efficient technique for the treatment of TN [14].

Aryan et al. [13] evaluated gamma knife and MVD for the treatment of TN and stated that MVD surgical technique would be advisable (if met with the surgery criteria) since complete recovery from the condition with the combined use of this technique and medication therapy was accomplished and 86% of patients attained relative recovery with the use of MVD technique [13]. This study also experienced that combination of medication and surgical therapy would achieve more reasonable results for the involved patients.

The results of a study by Hai et al. [21] on the efficacy of MVD surgical technique showed that precise evaluation and exact awareness of the location of involvement of blood vessels and nerves would result in a higher success rate of this treatment modality in patients with TN (80.8%).In the present study, of 14 patients undergoing MVD surgical technique, 64.28% achieved complete recovery, 14.8% had mild pain after surgery, and 21.42% had severe pain after surgery which is consistent with the results of the study performed by Hai et al. They stated that the application of MVD surgical technique in patients with TN removed the pressure on the nerve completely, resulting in complete pain relief in 50% of patients, relative pain relief in 30.8% of patients and mild pain relief or recurrence of pain in 19.2% of patients [21]. Aryan et al. reported a proper mean of response to MVD surgical procedure, with complete pain relief along with medication therapy which was inconsistent with the results yielded by the current study [13].

In a study performed by Oh et al. [22] the treatment outcomes of MVD and gamma knife radiosurgery were evaluated in adult patients and reported good prognosis in 17 patients (63%) in the MVD group with pain severity grades of I (no pain, no medication) and II (no pain with medication). The MVD procedure resulted in immediate pain relief in 70.3%, which decreased over time, showing evidently that the pain relief was achieved in long period [22].

Apfelboum carried out a study on the long-term outcomes of MVD in patients with TN and reported excellent treatment results (66%) and good results (15%) in patients with mild pain [23].

Regarding the outcomes of MVD in patients with TN, a study performed by Olson et al. demonstrated that 74% of patients achieved complete pain relief even 10 years after treatment [12].

In the present study, 69% of patients achieved complete or relative relief of pain and the severity of pain decreased in these patients. This result is consistent with the results of a study by Campbell et al. [19]. They evaluated the effect of Tegretol and placebo in patients with TN and showed that Carbamazepine could bring about a recovery and pain relief in 58% of patients in the first phase. Nevertheless, in their study placebo resulted in pain relief in 26% of patients, confirming the prominent effect of Carbamazepine in decreasing pain severity compared to placebo [19].

## Conclusion

Despite the availability of various treatment modalities for TN, approximately 30% of patients require continuance of their treatment or an alteration in their treatment modality. Moreover, medication therapy alone was less effective compared to medication therapy combined with surgical procedures. Even though both treatment modalities were significantly effective in the recovery of patients, further studies for evaluating the different modalities of treatments are strictly recommended. We would also recommend employing IMMACT guidelines [24] in future research for evaluation of chronic type of pain.
